# Integrative analysis of competing endogenous RNA networks reveals the functional lncRNAs in heart failure

**DOI:** 10.1111/jcmm.13739

**Published:** 2018-07-18

**Authors:** Zhimin Fan, Shanshan Gao, Yequn Chen, Bayi Xu, Chengzhi Yu, Minghui Yue, Xuerui Tan

**Affiliations:** ^1^ Department of Cardiology The First Affiliated Hospital of Shantou University Medical College Shantou Guangdong China; ^2^ Shantou University Medical College Shantou Guangdong China

**Keywords:** biological network, ceRNA, dysregulated ceRNA, heart failure, lncRNA

## Abstract

Heart failure has become one of the top causes of death worldwide. It is increasing evidence that lncRNAs play important roles in the pathology processes of multiple cardiovascular diseases. Additionally, lncRNAs can function as ceRNAs by sponging miRNAs to affect the expression level of mRNAs, implicating in numerous biological processes. However, the functional roles and regulatory mechanisms of lncRNAs in heart failure are still unclear. In our study, we constructed a heart failure‐related lncRNA‐mRNA network by integrating probe re‐annotation pipeline and miRNA‐target interactions. Firstly, some lncRNAs that had the central topological features were found in the heart failure‐related lncRNA‐mRNA network. Then, the lncRNA‐associated functional modules were identified from the network, using bidirectional hierarchical clustering. Some lncRNAs that involved in modules were demonstrated to be enriched in many heart failure‐related pathways. To investigate the role of lncRNA‐associated ceRNA crosstalks in certain disease or physiological status, we further identified the lncRNA‐associated dysregulated ceRNA interactions. And we also performed a random walk algorithm to identify more heart failure‐related lncRNAs. All these lncRNAs were verified to show a strong diagnosis power for heart failure. These results will help us to understand the mechanism of lncRNAs in heart failure and provide novel lncRNAs as candidate diagnostic biomarkers or potential therapeutic targets.

## 1 INTRODUCTION

Heart failure has become one of the top causes of death worldwide. Various cardiovascular diseases could increase the risks of heart failure, such as pathological cardiac hypertrophy, cardiac ischaemia and so on. Until now, a large number of studies have found that non‐coding RNAs (ncRNAs) play crucial roles in the biological processes of heart failure, implicating in activation of pathway or key protein. For example, SERCA2a gene therapy of heart failure restores miR‐1 expression by involving in Akt/FoxO3A‐dependent pathway, which is associated with normalized NCX1 expression and could improve cardiac function.^1^ Signalling cascades (calcineurin/Nfat) integrate with miR‐25 to induce the expression of the bHLH transcription factor Hand2 in the postnatal mammalian myocardium with impact on embryonic gene programmes in heart failure.^2^ Recently, a new class of ncRNA, long non‐coding RNA (lncRNA) has been a famous molecule in biology researches because of their abundant functions in complex biological regulation. LncRNAs, more than 200nt in length, are a subset of ncRNAs that can participate in various cellular processes, including genomic imprinting, RNA alternative splice, chromatin modification and post‐transcriptional regulation.^3^ Furthermore, lncRNAs have been demonstrated to implicate in pathology processes of multiple diseases.^4^

Importantly, more and more studies have found that lncRNA was a new regulatory factor in cardiovascular diseases.^5^ LncRNA Mhrt could antagonize the function of Brg1, a chromatin‐remodelling factor that is activated by stress to trigger aberrant gene expression and cardiac myopathy, protecting the heart from pathological hypertrophy.^6^ Klattenhoff et al found that lncRNA Braveheart (brvt) could function upstream of MesP1 to regulate a core cardiac gene network and interact with PRC2 to mediate epigenetic activation of the cardiac programme.^7^ However, it remains largely unknown as to how lncRNAs regulate cellular processes in heart failure.

Competing endogenous RNA (ceRNA) is a novel proposed mechanism, which elucidates the regulatory maps between miRNA targets in post‐transcriptional regulation levels.^8^ Interestingly, some studies have found this novel regulatory mechanism is occurred in multiple cardiovascular diseases. For instance, Wang et al found that lncRNA named CHRF acted as an endogenous sponge of miR‐489, which down‐regulated the expression level of miR‐489. CHRF is able to directly bind to miR‐489 and regulate Myd88 expression and hypertrophy.^9^ This team also demonstrated that lncRNA ARF could suppress miR‐188‐3p expression and then affect ATG7 expression, leading to autophagic cell death and myocardial infarction (MI).^10^ What's more, Song et al have proposed a pipeline to decode the cardiac hypertrophy‐associated ceRNA crosstalks between lncRNAs and mRNAs.^11^ Thus, it is necessary to investigate the essential regulatory mechanisms of lncRNAs in heart failure based on ceRNA. Large number of expression data could be downloaded from open database, such as GEO (https://www.ncbi.nlm.nih.gov/geo/). StarBase also provided the CLIP‐seq supported miRNA‐target interactions. All these data could enable us to achieve a global analysis to reveal the functional lncRNAs in heart failure.

**Figure 1:**
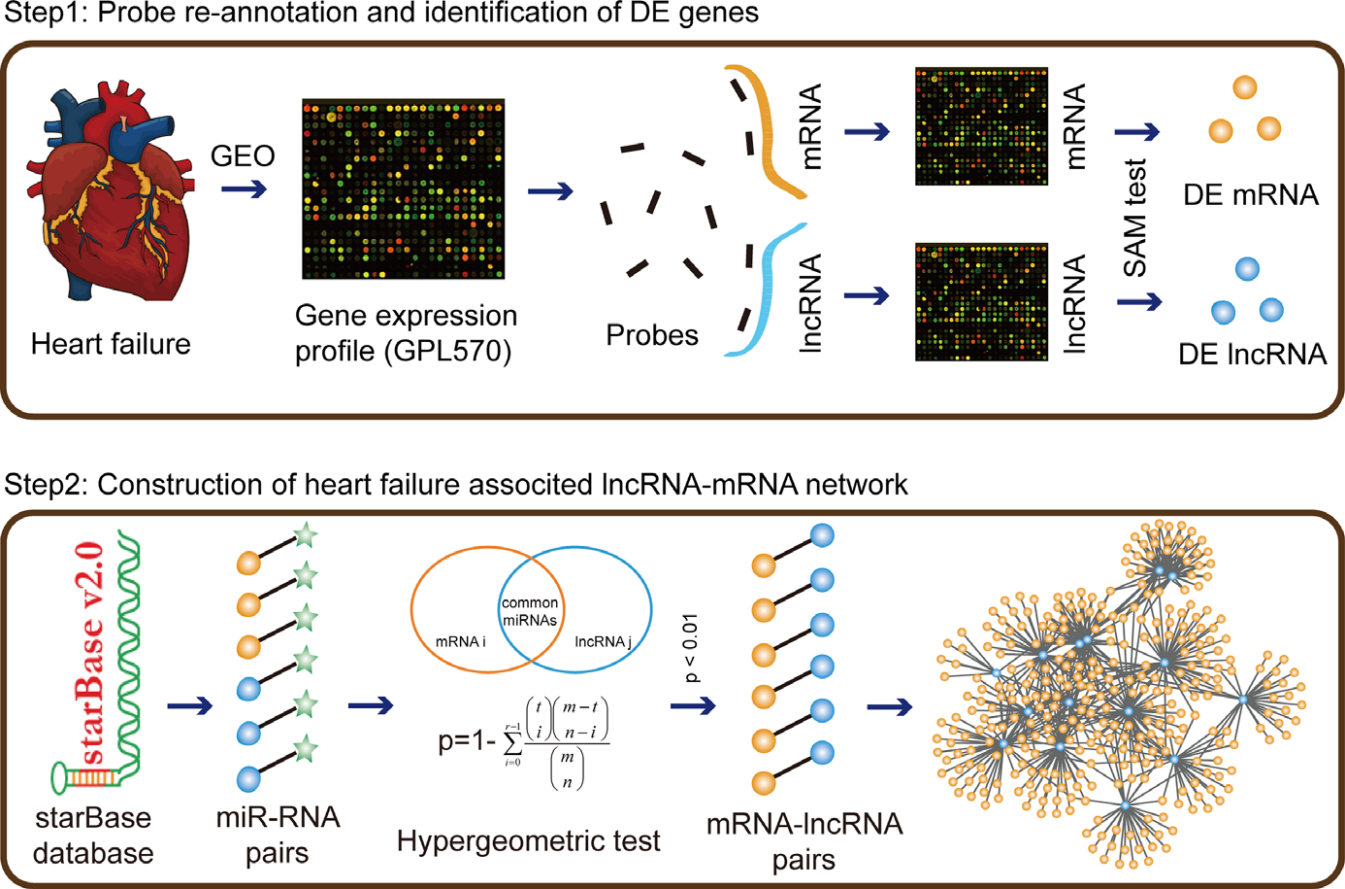
The pipeline for construction of heart failure‐related lncRNA‐mRNA network. First, we calculated the differentially expressed mRNAs and lncRNAs in normal and heart failure samples based on the re‐annotation results, using SAM test. We considered that lncRNA or mRNA with |fold change| >2 or *P*‐value < .01 as differentially expressed. Second, we mapped all these differentially expressed lncRNAs and mRNAs to the Ago CLIP‐supported miRNA‐target interactions and extracted the miRNA‐differentially expressed mRNAs interactions and miRNA‐differentially expressed lncRNA interactions. Third, we used hypergeometric test to identify lncRNA‐mRNA pairs at the threshold for *P*‐value < .01. Finally, all lncRNA‐mRNA pairs were merged to the heart failure‐related lncRNA‐mRNA network

In this study, to investigate the function of lnRNAs in heart failure, we constructed a heart failure‐related lncRNA‐mRNA network by integrating miRNA‐target interactions and differentially expressed information of genes/lncRNAs (Figure 1). First, we analysed topological features of network and identified several lncRNAs with important topological features. After performing bidirectional hierarchical clustering on the network, two modules that implicated in lncRNA‐mRNA interactions were identified. We found the two modules were enriched in heart failure associated pathways, suggesting lncRNA might exert their regulatory functions in regulating pathways. Moreover, based on the novel ceRNA mechanism, we calculated the Pearson correlation coefficients for all lncRNA‐mRNA interactions in normal and disease status, respectively. Thus, we could identify the dysregulated ceRNA interactions in normal or disease status, which could play a “switch” role in pathological processes.^12^ To predict more functional lncRNAs in heart failure, we performed a random walk algorithm on network by mapping known disease genes. We also validated the diagnosis power of our integrated 19 lncRNAs. In total, all these analyses and validations demonstrated how the functional lncRNAs regulated pathology processes in heart failure, and further suggested a potential therapeutics for heart failure.

## 2 MATERIALS AND METHODS

### 2.1 Gene expression profile

We downloaded the gene expression profile of heart failure with the accession number of GSE1145 from GEO database. GEO is a public functional genomics data repository supporting MIAME‐compliant data submissions. Array‐ and sequence‐based data are accepted. Tools are provided to help users query and download experiments and curated gene expression profiles. In our study, we analysed 26 biological samples that contained 11 control samples and 15 heart failure samples from platform GPL570. All samples were collected from patients undergoing cardiac transplantation whose failure arose from different aetiologies and from normal organ donors whose hearts cannot be used for transplants.

### 2.2 miRNA‐target interactions

Recently, study has found that crosslinking and Argonaute (Ago) immunoprecipitation coupled with high‐throughput sequencing (CLIP‐Seq) could help to identify endogenous interactions between miRNAs and their targets.^13^ starBase is designed for decoding Interaction Networks of lncRNAs, miRNAs, competing endogenous RNAs (ceRNAs), RNA‐binding proteins (RBPs) and mRNAs from large‐scale CLIP‐Seq (HITS‐CLIP, PAR‐CLIP, iCLIP, CLASH) data.^14^ In total, we download 423 975 miRNA‐mRNA interactions and 10 212 miRNA‐lncRNA interactions, including 13 861 mRNAs, 386 miRNAs and 1127 lncRNAs.

### 2.3 Probe re‐annotation pipeline

Studies found that re‐analysis of microarray probes could identify new transcripts’ expression data.^11^, ^15^ We also performed a probe re‐annotation pipeline to re‐analyse the microarray data to get both mRNA expression data and lncRNA expression data. Specifically, we downloaded probe sequences of platform GPL570 from Affymetrix (http://www.affymetrix.com) website. We then aligned these probe sequences to the human lncRNA transcript sequence and the human protein coding transcript sequences from GENCODE (http://www.gencodegenes.org/), using BLASTn. The alignment results were filtered by the following steps:

1. Only the probes that matched to one transcript were retained, the probes that matched to both the protein coding transcripts and lncRNA transcripts were removed, thus resulted in two sets of probes‐transcripts pairs.
2. In each set of probes‐transcripts pairs, removed the probes matched to more than one transcript.
3. Each transcript should be perfectly matched to more than three probes.

### 2.4 Construction of heart failure‐related lncRNA‐mRNA network

First, we calculated the differentially expressed levels of mRNAs and lncRNAs in normal and heart failure samples using SAM test, based on the re‐annotation results. We considered that lncRNA or mRNA with |fold change| >2 or *P*‐value < .01 as differentially expressed. Second, we mapped all these differentially expressed lncRNAs and mRNAs to the Ago CLIP‐supported miRNA‐target interactions and extracted the miRNA‐differentially expressed mRNAs interactions and miRNA‐differentially expressed lncRNA interactions. Third, we used hypergeometric test to identify lncRNA‐mRNA pairs at the threshold of *P*‐value < .01. The *P*‐value of a candidate lncRNA‐mRNA pair was measured as follows:

$$P\text{‐}\text{value}=1-\sum_{i=0}^{r-1}\frac{\left(\begin{array}{c} t\\ i \end{array}\right)\left(\begin{array}{c} m-t\\ n-i \end{array}\right)}{\left(\begin{array}{c} m\\ n \end{array}\right)}$$

where *m* represents the total number of miRNAs in starBase database, *t* represents the number of miRNAs that interacted with the mRNA, *n* represents the number of miRNAs that interacted with the lncRNA and *r* represents the number of miRNAs shared between mRNA and lncRNA.

The heart failure‐related lncRNA‐mRNA network was constructed by merging all these significant lncRNA‐mRNA pairs. Network was viewed by Cytoscape (http://www.cytoscape.org/).

### 2.5 Identification of lncRNA‐related modules in network

Many studies have demonstrated that lncRNAs tend to exert functions in a module.^16^, ^17^ To identify crucial lncRNA‐related modules in network, we performed a bidirectional hierarchical clustering and showed the result by R package “pheatmap.” In addition, gene ontology and pathway enrichment analysis were performed by DAVID (https://david.ncifcrf.gov/) and PATHWAX (http://pathwax.sbc.su.se/).

### 2.6 Identification of the dysregulated ceRNA interactions

To investigate the crucial role of dysregulated ceRNA interactions between normal and disease status, we developed a new computational pipeline by integrating lncRNA and mRNA expression levels in normal and disease samples. First, we calculated the Pearson correlation coefficients for all lncRNA‐mRNA interactions that were identified from previous step in normal samples and disease samples, respectively. All the lncRNA‐mRNA pairs with Pearson's correlation coefficients >0.5 in normal samples or disease samples were defined as ceRNA pairs. We used the change of expression correlation of the lncRNA‐mRNA pair in disease samples compared with normal samples to define the extent of dysregulation. Specifically, if the ceRNA pair's expression for disease samples shows a more positive correlation than for normal samples, the ceRNA pair is defined as “gain” dysregulation. If the ceRNA pair's expression for normal samples shows a more positive correlation than for disease samples, the ceRNA pair is defined as “loss” dysregulation. And the change of correlation was defined as follows:

$$\Delta R={\text{Corr}}_{\mathrm{disease}}\left(A,\;B\right)-{\text{Corr}}_{\mathrm{normal}}\left(A,\;B\right)$$

where Corr_disease_(A, B) represents the Pearson correlation coefficient between gene A and gene B in disease samples, Corr_normal_(A, B) represents the Pearson correlation coefficient between gene A and gene B in normal samples.

To obtain the statistically significant Δ*R* of each ceRNA pair, we performed 1000 times permutation for each ceRNA pair by randomly permuting normal/disease sample labels. The significant *P*‐value of each Δ*R* was given as the frequency of the Δ*R* values in random conditions, which was greater than the Δ*R* value in the real condition. We only reserved the ceRNA pairs with Δ*R* > 0.5 or *P*‐value < .05 for further analysis.

### 2.7 Random walk with restart on heart failure‐related lncRNA‐mRNA network

A random walk in network was defined as an iterative walker's transition from a certain node to a randomly selected neighbour that started from a given node (Node *i*). In this study, random walk that we performed had capacity of restart with probability *r* in every time step at “Node *i*.” The random walk with restart was defined as follows:

$$p^{t+1}=\left(1-r\right)Wp^{t}+rp^{0}$$

where *W* represents the column‐normalized adjacency matrix of the network, *p* ^*t*^ is a vector whose size is equivalent to the number of nodes in the network and the *i*‐th element holds the probability of being at node *i* at time step *t*.

In our study, the initial probability vector *p* ^0^ was constructed as follows: 1 was assigned to the nodes that represented known disease genes from DisGeNET, and 0 was assigned to the other nodes. We considered that the role of disease genes was equivalent in network. Vector *p* would be in the steady‐state at time step *t* where *t* approached infinity as a limit. The iteration would be finished till the change between *p* ^*t*^ and *p* ^*t*+1^ fell below 10^−10^.

In addition, we performed statistical significance analysis for the scores of each lncRNA that yielded from random walk. The statistical significance for rejection of the null hypothesis was determined by comparing the scores of lncRNAs in the network following *n* iterations of that known disease genes shuffling. To strictly keep network topological properties, random sampling without replacement was performed when doing the random disturbance, and the degree distribution was guaranteed the same between selection seed node and the real. When iterating, the times that the score of every lncRNA was higher than the real one was record as *m*. The statistical significance *P*‐value for every lncRNA was calculated by the ratio of *m* and *n*. In this work, *n* was set at 3000 times.

### 2.8 Identification of diagnostic biomarkers

To evaluate the diagnosis power of the dysregulated lncRNAs, the scoring classifier was constructed. For each lncRNA, we first performed *Z*‐score transformation on the expression levels across the samples for each lncRNA and then summarized the *Z*‐scores as the integrated expression signature. Then, the samples would be divided into two classes (control and heart failure) by choosing a cut‐off. And the receiver operation characteristic (ROC) curve was used for classifier evaluation which was drawn by plotting sensitivity against the false‐positive rate. This procedure was performed using the R package pROC. In addition, we selected same number of lncRNAs as random biomarkers to perform the last step.

## 3 RESULTS

### 3.1 Identification of differentially expressed lncRNAs and genes

To identify the differentially expressed lncRNAs and genes in heart failure, we downloaded the gene expression profile from GEO database. According to previous studies, we decided to use a probe re‐annotation strategy to detect lncRNA and gene expressions. In our study, we re‐annotated the probe annotation data for platform GPL570 that is developed by Affymetrix. Specifically, probe sequences were downloaded from Affymetrix. Using the alignment tool Blastn to alignment the sequences between probes and lncRNAs/genes, the probe‐gene, probe‐lncRNA pairs were annotated. As a result, we re‐annotated ~14 000 probe‐gene pairs and ~4000 probe‐lncRNA pairs for platform GPL570.

We downloaded the heart failure associated gene expression profile on platform GPL570 (accession number GSE1145), which contained more than 100 samples with failure arising from different aetiologies. We only retained 11 control samples and 15 heart failure samples for analysis. Based on the re‐annotation results, we performed SAM test to identify the differentially expressed genes and lncRNAs. Totally, 1081 differentially expressed genes and 993 differentially expressed lncRNAs were identified as the potential regulators in dysfunction heart at the threshold of |Fold change|>2 or *P*‐value < .01.

### 3.2 Construction of heart failure related lncRNA‐mRNA network

**Figure 2:**
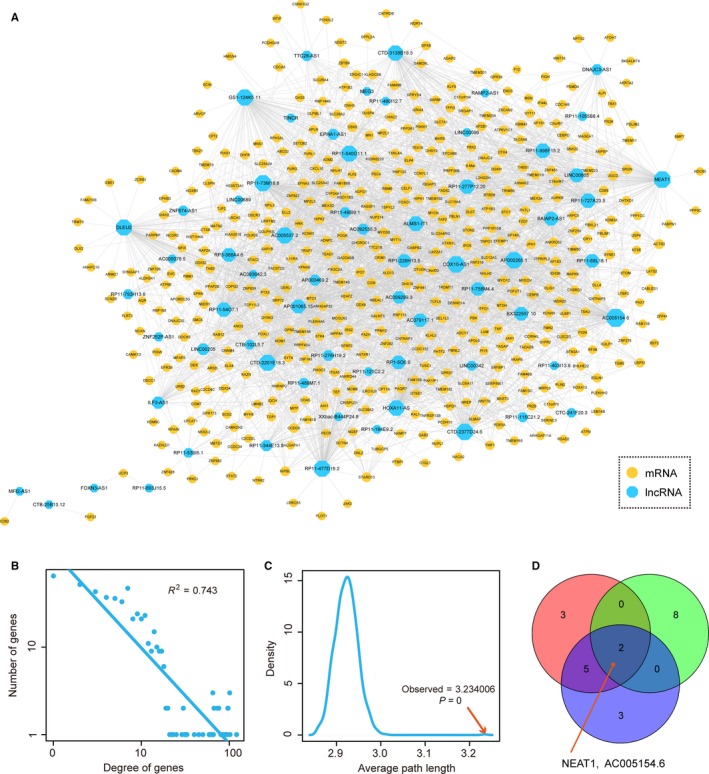
Topological features of heart failure‐related lncRNA‐mRNA network. A, The view of heart failure‐related lncRNA‐mRNA network. Blue nodes represented lncRNAs and yellow nodes represented mRNAs. Node size represented degree. B, Degree distributions of the network. All degrees followed a power‐law distribution. C, Average path length distributions of the real network and 1000 times random networks. Average path length in real network was larger than in random cases. D, The distributions of top 10 nodes of degree, betweenness and closeness

To construct the heart failure‐related lncRNA‐mRNA network, we integrated the differentially expressed lncRNAs/mRNAs and the Ago‐CLIP supported miRNA‐target interactions. First, we downloaded the Ago‐CLIP supported miRNA‐mRNA and miRNA‐lncRNA interactions from the starBase database, which included 423 975 miRNA‐mRNA pairs and 10 212 miRNA‐lncRNA pairs. Then, we mapped differentially expressed lncRNAs and mRNAs into the miRNA‐lncRNA pairs and miRNA‐mRNA pairs, respectively. We extracted differentially expressed lncRNAs/mRNAs‐related miRNA‐lncRNA/mRNA interactions for further analysis. To identify the lncRNA‐mRNA pair, we listed all the candidate lncRNA‐mRNA pairs by sharing the common miRNAs. The measure of hypergeometric test was performed to calculated statistical significances for all candidate lncRNA‐mRNA pairs. Finally, we extracted 2944 candidate lncRNA‐mRNA pairs with *P*‐value < .01 and merged all these pairs into the heart failure‐related lncRNA‐mRNA network, including 66 lncRNA nodes and 451 mRNA nodes (Figure 2A).

### 3.3 Topological feature analysis of the heart failure‐related lncRNA‐mRNA network

After constructing the heart failure‐related lncRNA‐mRNA network, we then performed network topological analysis. Analysis of biological network could identify many crucial regulators that played key roles in biological processes. First, we performed network degree analysis. Results showed that degrees of all nodes followed a power‐law distribution (Figure 2B), suggesting the network was scale‐free, similar to most of biological networks. In this network, a small subset of nodes, denoted as hubs, linked many interacting partners. Moreover, we calculated average path length for the network, and results showed that the characteristic path length of the network was substantially larger than that of random networks (1000 times random network, *P* < .001) (Figure 2C), indicating that the network had reduced global efficiency. These results suggested that dysregulated lncRNA‐mRNA interactions often occurred in a local scale and hubs tended to be critical in entire network. In addition, studies found that nodes occupied crucial topological features in biological network often played a more important regulatory role in biology. Thus, we further calculated betweenness and clossness for the nodes in network, respectively. For every pair of nodes in a network graph, there exists at least one shortest path between the nodes. Betweenness for each node is defined as the number of the shortest paths that pass through the node. Closeness of a node is a measure of centrality in a network, calculated as the sum of the length of the shortest paths between the node and all other nodes in the network graph. For the three kinds of topological features, top 10 crucial nodes were selected (Table S1). Interestingly, we found two lncRNAs (NEAT1 and AC005154.6) were all crucial in each feature (Figure 2D), indicating that the two lncRNA might function as a crucial regulators in the pathology processes of heart failure. NEAT1 is the lncRNA that localizes to hundreds of genomic sites in cells, primarily over active genes.^18^ Some studies had found that NEAT1 functioned in multiple biological processes, such as altering the epigenetic landscape of target gene promoters, regulating cell proliferation and implicating in miRNA‐associated pathways.^19^, ^20^, ^21^ Heart failure always accompanied with myocardial fibroblasts proliferation. Studies have demonstrated that miRNA‐associated pathways were highly related to heart failure.^1^

### 3.4 Module analysis of heart failure‐related lncRNA‐mRNA network

**Figure 3:**
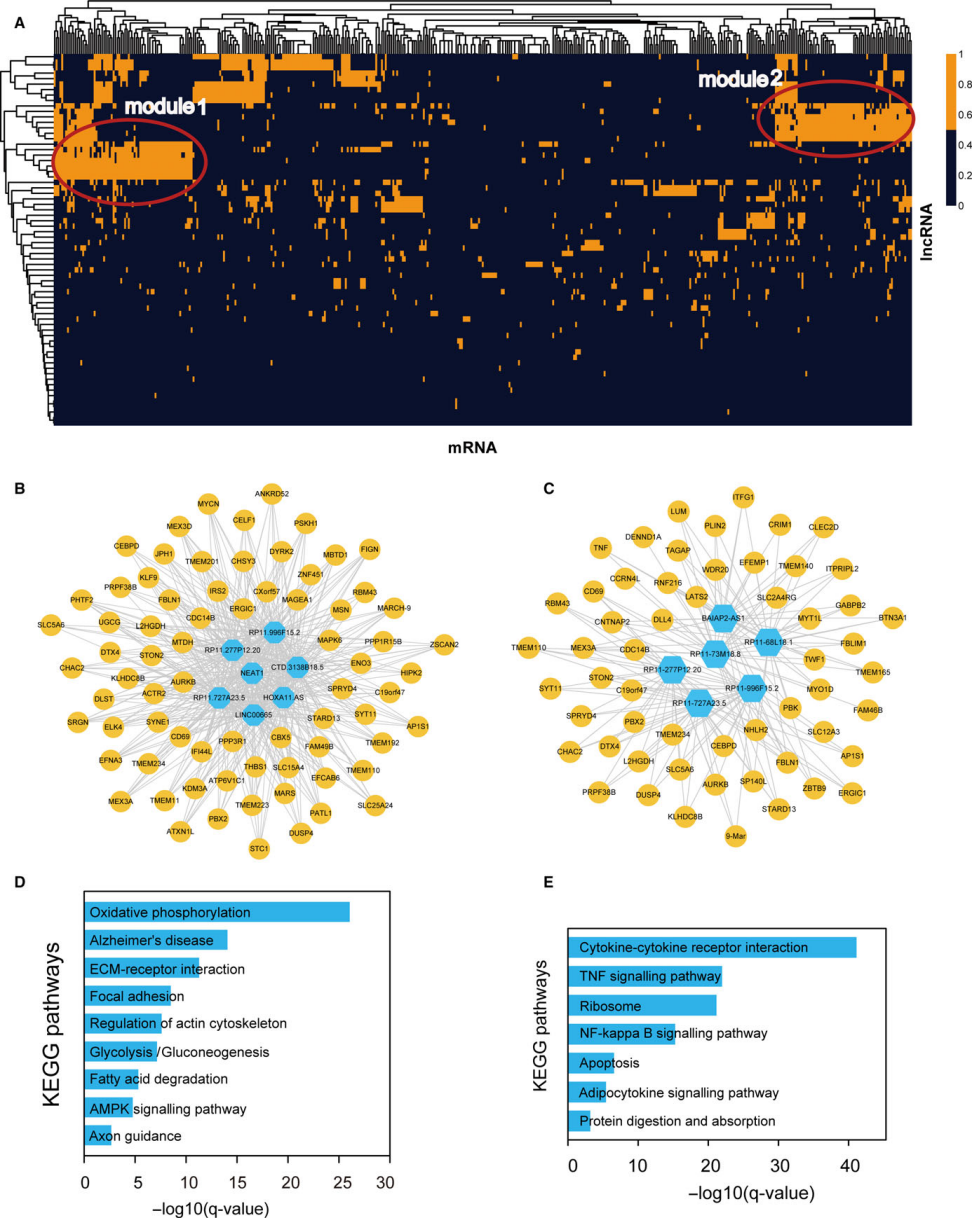
Module analysis of heart failure‐related lncRNA‐mRNA network. A, Bidirectional hierarchical clustering of heart failure‐related lncRNA‐mRNA network. The clusters in red circle represented two modules. B, The lncRNA‐mRNA interactions in module 1. C, The lncRNA‐mRNA interactions in module 2. D, Pathway enrichment of mRNAs in module 1. E, Pathway enrichment of mRNAs in module 2

Many studies have demonstrated that lncRNA often exerted functions by involving in functional modules. Thus, we performed module analysis to identify the lncRNA‐associated functional modules from the heart failure‐related lncRNA‐mRNA network, using bidirectional hierarchical clustering (Figure 3A). In the heat map, we identified two modules highly clustered, implying that some lncRNAs and mRNAs could form a complex module to function in heart failure (Figure 3A). We extracted the corresponding lncRNAs and mRNAs in these two dense modules (Figure 3B,C). Pathway analysis was performed on the genes in module 1. Results showed that these genes were enriched in some heart failure‐related pathways, such as ECM‐receptor interaction, Focal adhesion and AMPK signalling pathway (Figure 3D). Volume overload‐induced heart failure results in progressive left ventricular remodelling characterized by chamber dilation, eccentric cardiac myocyte hypertrophy and changes in extracellular matrix (ECM) remodelling changes.^22^ ECM accumulation is common in multiple cardiovascular diseases, leading to heart failure. Focal adhesion kinase activation could be important for the adaptive response to increase in cardiac afterload by controlling the activity of PI3K/AKT/mTOR pathway.^23^ AMP‐activated protein kinase (AMPK) is activated when intracellular ATP production decreases. AMPK has critical roles in regulating growth and reprogramming metabolism and has recently been connected to cellular processes such as autophagy and cell polarity.^24^ Activation of AMPK significantly improves left ventricular function and survival in heart failure.^25^

Similarly, pathway analysis was also performed on the genes in module 2 (Figure 3E). Results showed that they were enriched in some heart failure‐related pathways, such as tumour necrosis factor (TNF) signalling pathway, NF‐kappa B signalling pathway and Apoptosis. TNF is a crucial regulator in heart failure. Previous study has found that TNF‐alpha contributed to the myocardial remodelling process in evolving heart failure through the local induction of specific myocardial matrix metalloproteinases.^26^ Gupta et al found that inhibition of NF‐κB could reduce the expression of atrial natriuretic factor to prevent cardiac hypertrophy and heart failure.^27^ Persistent myocyte NF‐κB p65 activation in heart failure could exacerbate cardiac remodelling by imparting pro‐inflammatory, pro‐fibrotic and pro‐apoptotic effects.^28^ In addition, apoptosis is also a crucial cellular process in the pathology of heart failure.^29^, ^30^ These results indicated that lncRNAs could exert their regulatory roles by implicating in gene modules and effectively regulating downstream pathways of heart failure.

### 3.5 Identification of dysregulated ceRNA interactions in network

**Figure 4:**
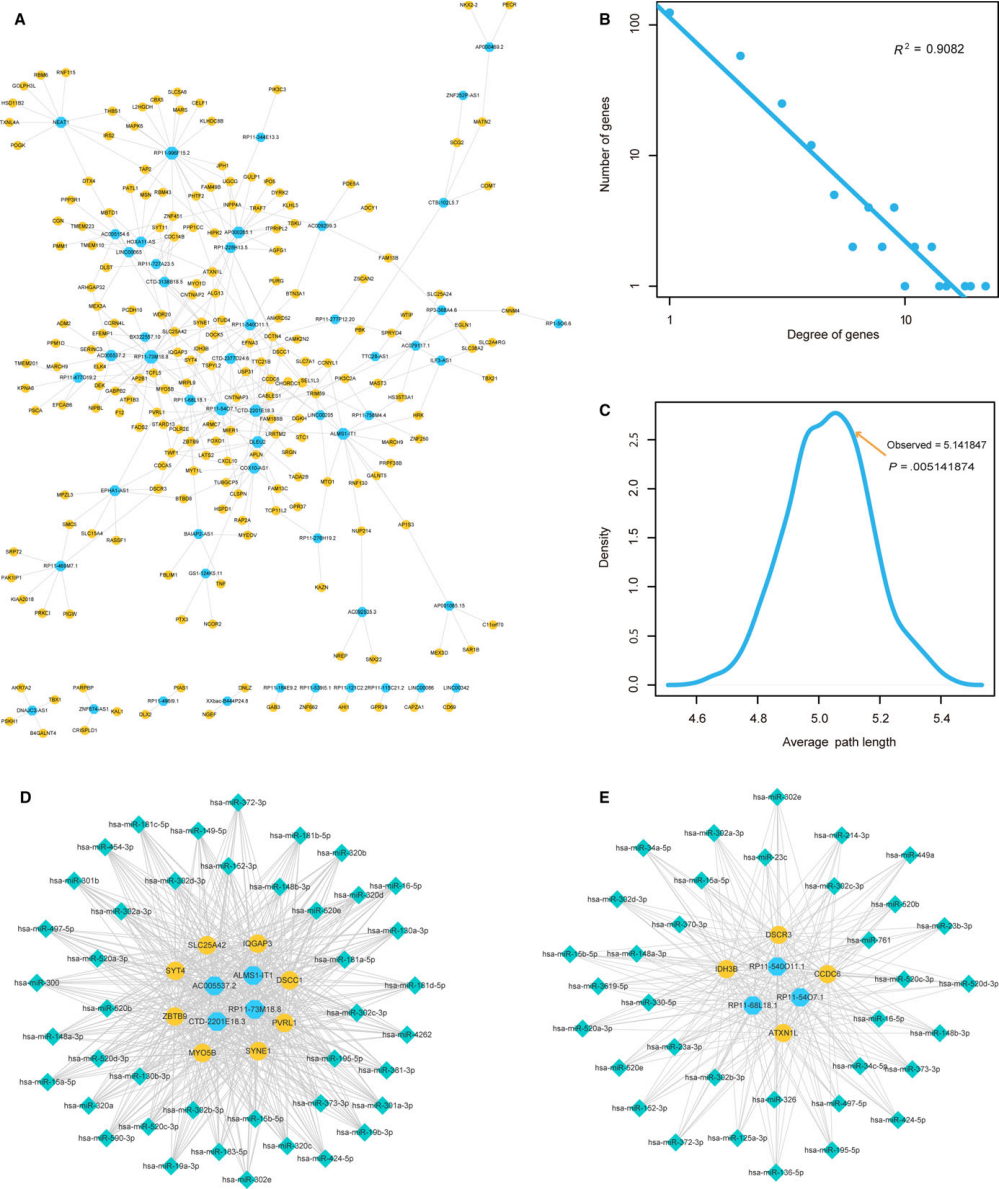
Dysregulated ceRNA interactions in heart failure. A, The view of dysregulated ceRNA network. Blue nodes represented lncRNAs and yellow nodes represented mRNAs. B, Degree distributions of the network. C, Average path length distributions of the real network and 1000 times random networks. D, Hub‐associated ceRNA interactions in “gain” module. E, Hub‐associated ceRNA interactions in “loss” module

Dysfunction of ceRNA interactions has been demonstrated to be a crucial regulatory process in pathology of diseases. To identify the dysregulated ceRNA interactions in network, we performed analysis as follows: (i) Pearson's correlation coefficients were calculated for all lncRNA‐mRNA interactions in normal samples and heart failure samples, respectively. Those lncRNA‐mRNA pairs with Pearson's correlation coefficients >0.5 in normal samples or disease samples were defined as ceRNA pairs; (ii) the changes in Pearson's correlation coefficients between normal and disease conditions (“gain” or “loss” dysregulation) were calculated; (iii) 1000 times permutations for step i were performed to identify the statistical significant dysregulated ceRNA pairs. We only retained the ceRNA pairs with Δ*R* > 0.5 or *P*‐value < .05 as the dysregulated ceRNA pairs (see in Methods). As a result, 135 “gain” ceRNA pairs and 178 “loss” ceRNA pairs were identified as dysfunctional in heart failure for further analysis. We merged all these dysregulated ceRNA pairs into the heart failure‐related dysregulated ceRNA network (Figure 4A).

We analysed the topological features of the dysregulated ceRNA network. All nodes in network followed a power law distribution (Figure 4B), suggesting the network was scale‐free. Average path length in this network was larger than random networks (Figure 4C). All these results encouraged us to investigate the dysregulated ceRNA interactions in local scales. Hubs were demonstrated to play key roles in biological processes. We investigated the functions of hubs in “gain” ceRNA interactions and “loss” ceRNA interactions, respectively. For example, we merged all the “gain” ceRNA interactions into network and selected those mRNA/lncRNA nodes with the top 10% degrees as hubs. All ceRNA interactions among hubs were extracted to constitute a “gain” module (Figure 4D). “Loss” module was also identified by the same pipeline (Figure 4E). In addition, we extracted miRNAs that mediated ceRNA interactions to elucidate the importance of dysregulated ceRNA in heart failure. In “gain” module, we found some heart failure‐related miRNAs were involved in the regulation processes of ceRNA interactions. For example, miR‐181c targeted to 3′UTR of the mRNA of a mitochondrial gene, mt‐COX1. Overexpression of miR‐181c could decrease mt‐COX1 protein expression, increasing production of reactive oxygen species in heart failure.^31^ Mir‐148 was a repressor of NF‐kB signalling, playing roles in cardiac injury.^32^ In addition, miR‐19 was demonstrated to involve in cardiac remodelling by controlling cardiac fibroblast proliferation and migration.^33^ Moreover, in “loss” module, a number of heart failure‐related miRNAs were also extracted. For instance, miR‐34 family was demonstrated to attenuate pathological cardiac remodelling and improve heart function. Inhibition of miR‐34 could reduce cardiac fibrosis, increase angiogenesis, increase Akt activity, decrease atrial natriuretic peptide (ANP) gene expression and maintain sarcoplasmic reticulum Ca^2+^ ATPase gene expression.^34^ Inhibition of miR‐15 family could reduce infarct size and cardiac remodelling and enhance cardiac function in response to MI.^35^ Huang et al demonstrated that overexpression of miR‐16 could provoke cardiomyocyte hypertrophy by derepressing the cyclins D1, D2 and E1, and activating cyclin/Rb pathway.^36^ These results suggested that miRNA‐mediated dysregulated ceRNA interactions played important roles in pathology of heart failure.

### 3.6 Identification of heart‐related lncRNAs based on random walk

To identify more heart failure‐related lncRNAs based on the heart failure‐related lncRNA‐mRNA network, we performed a random walk algorithm to the network. First, we downloaded all the known disease genes of heart failure from DisGeNET (http://www.disgenet.org/web/DisGeNET/), which is a platform that integrates information on gene‐disease associations from several public data sources and the literatures. We mapped 16 disease genes into the network and scored for lncRNA nodes by random walking. To obtain the significances for scores of lncRNAs, we performed 3000 time permutations and only reserved the lncRNAs with the *P*‐value < .05. As a result, we found three significant lncRNAs (Table S2), which were considered important in the pathology processes of heart failure. Additionally, we also extracted their miRNA interaction partners. We found that miR‐370 targeted to both RP11‐693J15.5 and FOXN3‐AS1. MiR‐370 was demonstrated to affect many signalling pathways, such as Akt/FoxO3a,^37^ TGFβ signalling pathway.^38^ All these pathways were crucial in the regulatory processes of heart failure.

### 3.7 Diagnosis power of dysregulated lncRNAs in heart failure

**Figure 5:**
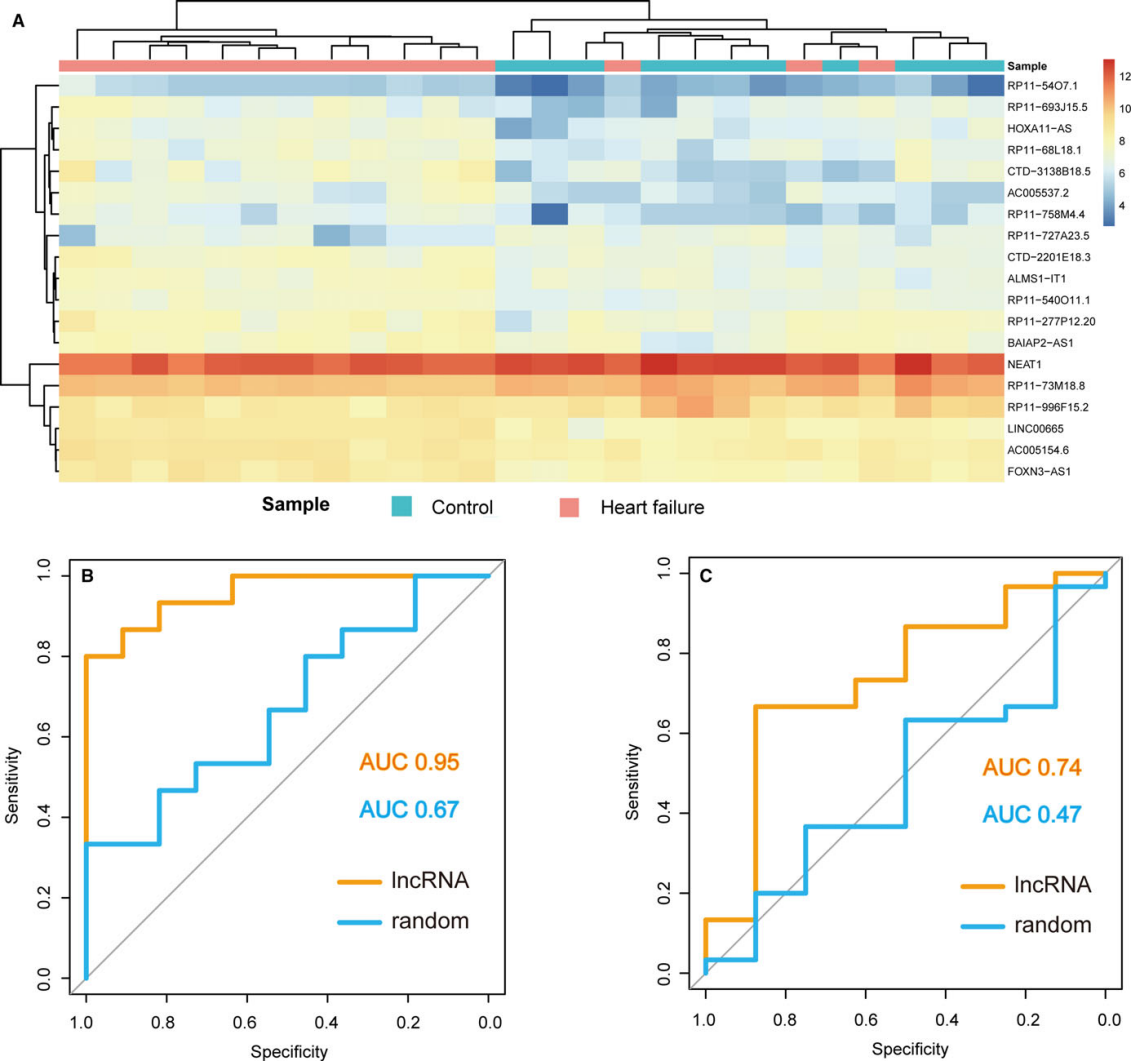
Diagnosis power of lncRNAs. A, Unsupervised hierarchical clustering for the expressions of 19 lncRNAs that identified from previous steps. B, The discriminatory performance of the 19 lncRNAs in GSE1145 data set, evaluated by ROC analysis and calculating the AUC. C, The discriminatory performance of the 19 lncRNAs in GSE21610 data set, evaluated by ROC analysis and calculating the AUC

To identify the diagnosis power of biomarkers in heart failure, we performed unsupervised hierarchical clustering for the expressions of 19 lncRNAs, which were obtained from previous steps. Results showed that all samples were grouped into two distinctive sample clusters (11 samples in Cluster 1, 15 samples in Cluster 2), which were highly correlated to disease status. In Figure 5A, all control samples were grouped into Cluster 2, most heart failure samples (12/15, 80%) were grouped into Cluster 1. The result indicated that these 19 lncRNAs could be used as biomarkers in the diagnosis of heart failure. Thus, we integrated these 19 lncRNAs to form a module (termed lncRNA). The performance of the module was evaluated by the area under the ROC curve (AUC). ROC curves of the module gave AUC values of 0.95 in the GSE1145 data set, which was larger than the random case (Figure 5B). In addition, we used the other independent data set of dilated cardiomyopathy (GSE21610) that downloaded from GEO database to evaluate the diagnosis effect of the lncRNA modules. Results showed that the lncRNA module also produced a strong diagnosis effect on the early heat failure (Figure 5C). All these results proposed a novel clinical application of lncRNAs in heart failure.

## 4 DISCUSSION

Heart failure is a common outcome of various cardiovascular diseases, such as cardiac hypertrophy, myocardial infarction and has become a leading cause of death worldwide. Thus, it is essential to discover the effective therapeutic targets for heart failure. During the past years, great efforts have been made to provide the novel insights into the molecular mechanisms for decoding heart failure. But more studies have focused on elucidating the molecular mechanism that involved in protein‐coding genes and miRNAs.^39^ With the rapid development of next‐generation sequencing, a new type of long non‐coding transcripts that termed lncRNAs and encompassed more than 200 nucleotides have been discovered. This novel transcript has been demonstrated to play important roles in wide range of biological processes.^40^ Dysfunction of lncRNAs could lead to the changes in various complicated disease phenotypes, from cancer to cardiovascular disease.^41^, ^42^ It has become a necessary way to investigate the underlying mechanisms of lncRNAs in heart failure for clinical therapy. Most of all, there is increasing evidence that lncRNAs can be targeted by miRNAs to exert functions as ceRNAs.^43^ LncRNAs can affect the expression of protein coding genes through competitively binding to shared miRNAs, reducing the degradation of protein coding genes. In cardiovascular disease, several studies have found that the lncRNA‐associated ceRNA crosstalks are crucial in the pathology processes. However, there is no systematical analysis for lncRNA‐associated ceRNA crosstalks in heart failure to reveal the functional lncRNAs.

In this study, we performed a computational analysis to investigate the function of lncRNAs in heart failure. We constructed a heart failure‐related lncRNA‐mRNA network through integrating Ago CLIP‐seq supported miRNA‐target interactions and differentially expressed information of genes/lncRNAs. First, we performed network topological analysis and identified several lncRNAs with important topological structures. After performing bidirectional hierarchical clustering, two modules that contained more lncRNA‐mRNA interaction clusters were identified from the network. We found the two modules were enriched in pathways that were highly related to heart failure, suggesting lncRNA might exert their regulatory functions by implicating in pathways. Moreover, based on the novel ceRNA mechanism, we calculated the Pearson correlation coefficients for all lncRNA‐mRNA interactions in normal and disease status, respectively. We identified the dysregulated ceRNA interactions in normal or disease status, which could play a “switch” role in pathological processes.^22^ Additionally, to predict more functional lncRNAs in heart failure, we performed a random walk algorithm on network by mapping known disease genes. Results showed that all these lncRNAs had a strong diagnosis power and could be used as biomarkers in heart failure.

Importantly, we investigated the dysregulated ceRNA interactions that might play “switch” role in heart failure. Recent studies have demonstrated that ceRNAs exerted functions in certain disease or physiological status. Paci et al constructed normal and cancer networks of miRNA mediated lncRNA‐mRNA interactions, using breast cancer expression data.^44^ Li et al identified the change of ceRNA networks in different subtypes of prostate cancer based on a previously predicted ceRNA network.^45^ In this study, we constructed a dysregulated ceRNA network by integrating the miRNA‐target interactions and differentially expressed information of genes/lncRNAs. The network was scale‐free and had a higher average path length than random network. Furthermore, we emphatically analysed the hub nodes in “gain” module and “loss” module. MiRNAs that mediated dysregulated ceRNA interactions were extracted, which have been demonstrated to play crucial roles in the pathology of heart failure. For example, overexpression of miR‐181c could decrease mt‐COX1 protein expression, increasing production of reactive oxygen species in heart failure.^31^ MiR‐34 family was demonstrated to attenuate pathological cardiac remodelling and improve heart function. Inhibition of miR‐34 could reduce cardiac fibrosis, increase angiogenesis, increase Akt activity, decrease ANP gene expression and maintain sarcoplasmic reticulum Ca^2+^ ATPase gene expression.^34^ All these results can help us better understand the initiation and propagation of heart failure.

However, our study also had some limitations. First, we used a probe re‐annotation pipeline to identify heart failure‐related functional lncRNAs, which has been widely used by many bioinformatics studies. However, we had to admit this pipeline filtered out many lncRNAs that miss matched to the probe sequences. Recently, because of the insufficient open data resource, we could not obtain more valid data. Like the microarray platform GPL570, only a few studies and biological samples focused on heart failure. This probe re‐annotation pipeline could indeed help researchers re‐analyse the microarray data to get both mRNA expression data and lncRNA expression data. In future, with the next‐generation sequencing data increasing, we would add more heart failure‐related lncRNA data information for more detailed analysis. Second, we integrated gene expression and miRNA‐target interactions to identify dysregulated ceRNA interactions. If a more accurate algorithm was proposed, our results will be more stable. Third, as a research in the bioinformatics field, this work aimed at verifying accuracy and reliability of the lncRNA‐mRNA network, the lncRNA‐associated functional modules or the diagnostic potentiality of the biomarker lncRNAs often through the way of statistical significances and scientific literature validation. In future studies, if conditions allowed, we would add experimental design to perform more detailed mechanism research and further validate our conclusions. However, to validate the expression of risk lncRNAs in heart failure, we further downloaded another six heart failure‐related gene expression data sets from GEO database and performed the up/down‐regulated analysis for lncRNAs. Results demonstrated that most of the 19 lncRNAs showed a similar expression trend in at least three datasets (Table S3), indicating that the 19 risk lncRNAs had a stable expression pattern in heart failure.

In summary, we performed a network analysis to identify functional lncRNAs by multiple dimensions in heart failure. First, we identified the lncRNAs with the central topological features. Second, bidirectional hierarchical clustering identified some lncRNAs functioned in modules. Third, we identified the lncRNA‐associated dysregulated ceRNA interactions. And we performed a random walk algorithm to identify more heart failure‐related lncRNAs. Additionally, all these lncRNAs showed a strong diagnosis power for heart failure. These results will help us to understand the mechanisms of lncRNAs in heart failure and provide novel lncRNAs as candidate diagnostic biomarkers or potential therapeutic targets.

## CONFLICTS OF INTEREST

The authors declare that they have no conflicts of interest to disclose.
